# Supplementary figures and images for: Gonococcal invasion into epithelial cells depends on both cell polarity and ezrin
Source: PLoS Pathog. 2021 Dec 1;17(12):e1009592. doi: 10.1371/journal.ppat.1009592 (PMC8668114; doi:10.1371/journal.ppat.1009592)

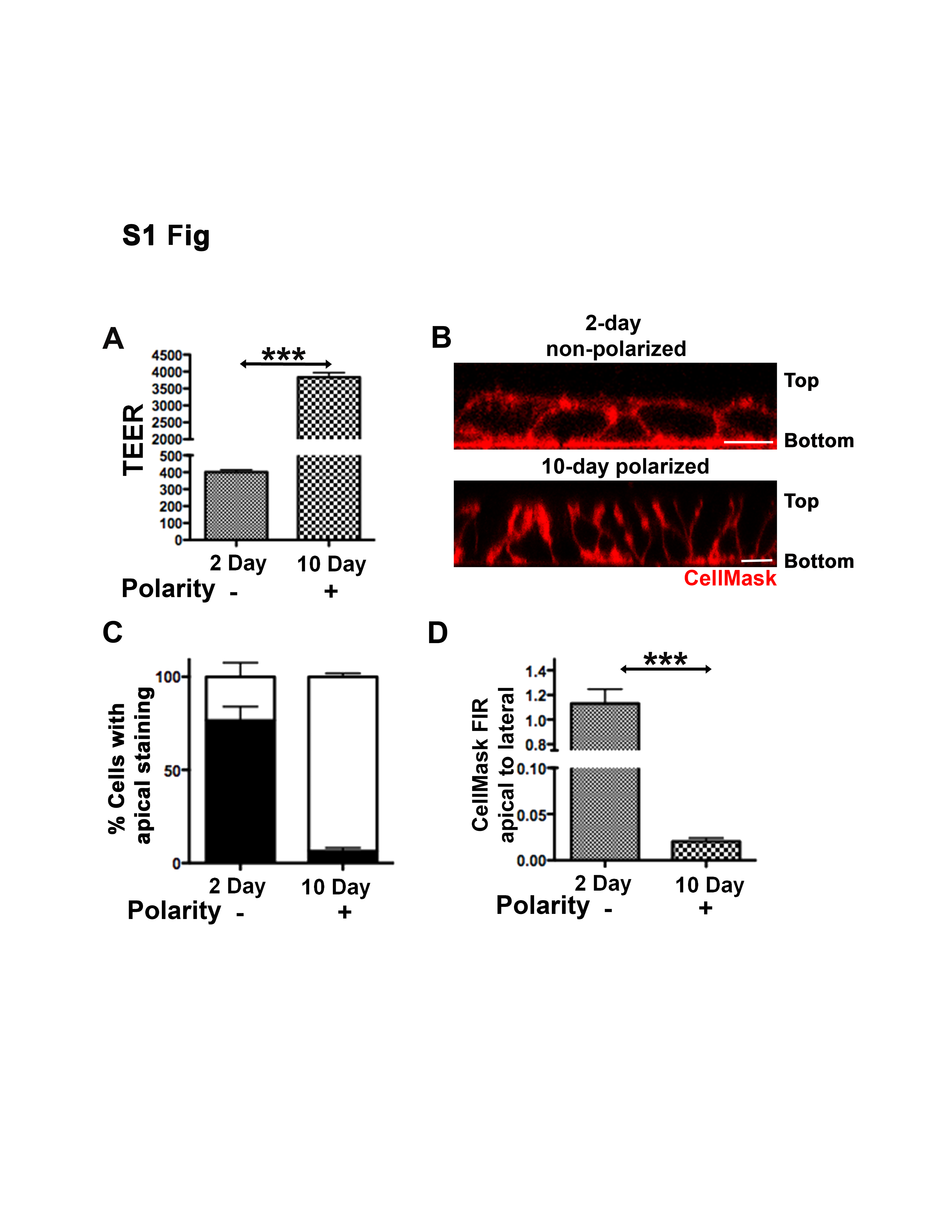

Supplement: S1 Fig — T84 cells were cultured on transwells for 2 or 10 days. (A) Transepithelial electric resistance (TEER) indicating the ion permeability of the epithelium. (B-D) The 2- and 10-day T84 cells were cultured with the CellMask lipid dye in the bottom chamber for 15 min and imaged using a confocal fluorescence microscope (CFM). Shown are representative xz images (B), the percentage of cells with (black) and without (white) CellMask dye diffusion from the bottom into the top chamber (C), and the fluorescence intensity ratio (FIR) of CellMask staining at the top to the bottom membrane of T84 cells (D). n = 3 three independent experiments. ***p<0.001, Scale bar, 10 μm. (TIF) [file ppat.1009592.s001.tif]

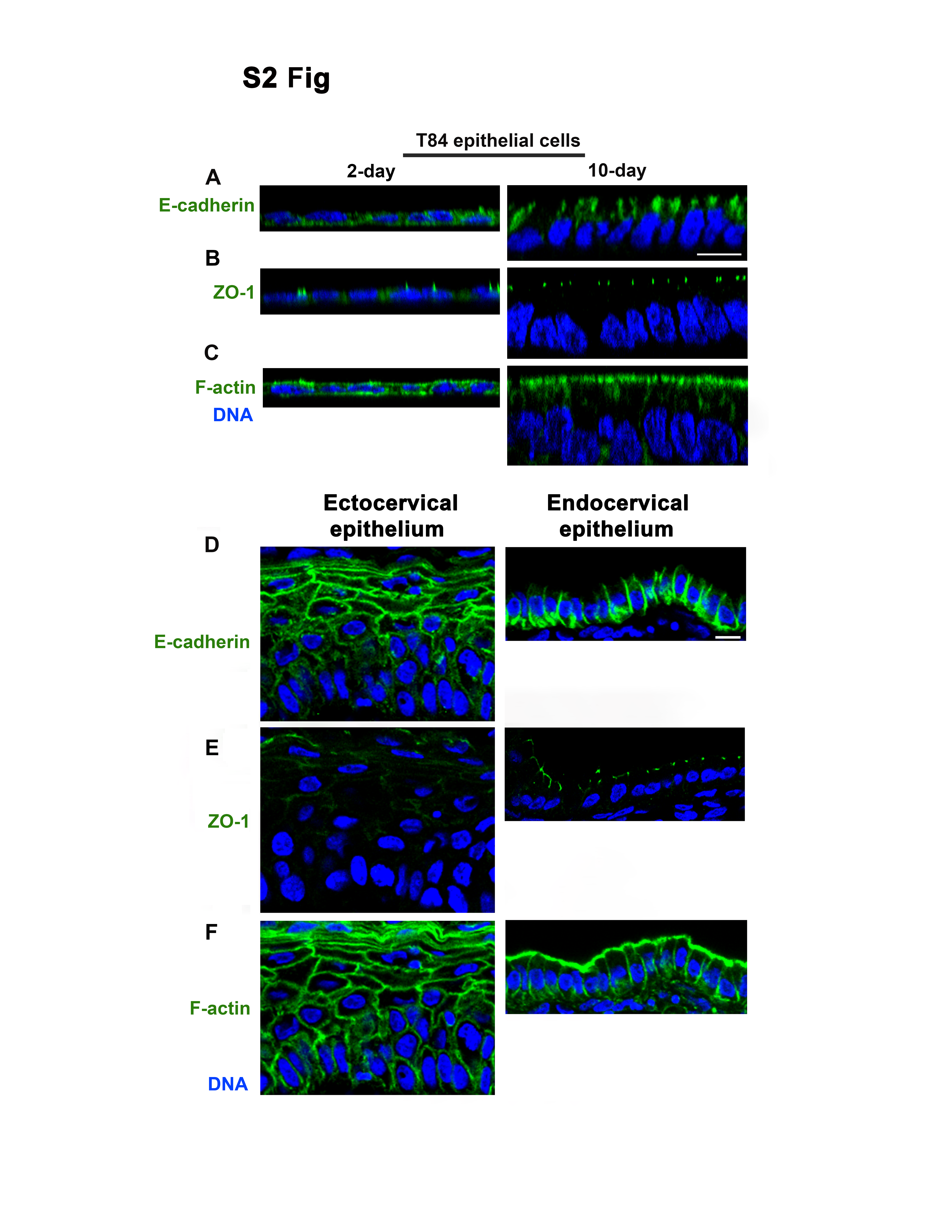

Supplement: S2 Fig — (A-C) T84 cells were cultured on transwells for 2 or 10 days, stained for E-cadherin (A), ZO-1 (B), F-actin (C), and Hoechst, and imaged by a CFM. Shown are representative xz images. (D-F) Human cervical tissue explants were cultured for two days, fixed, and cryopreserved, sectioned, stained for E-cadherin (D), ZO-1 (E), F-actin (F), and Hoechst, and imaged using a CFM. Shown are representative images across epithelia. n = 4 four independent experiments. Scale bar, 10 μm. (TIF) [file ppat.1009592.s002.tif]

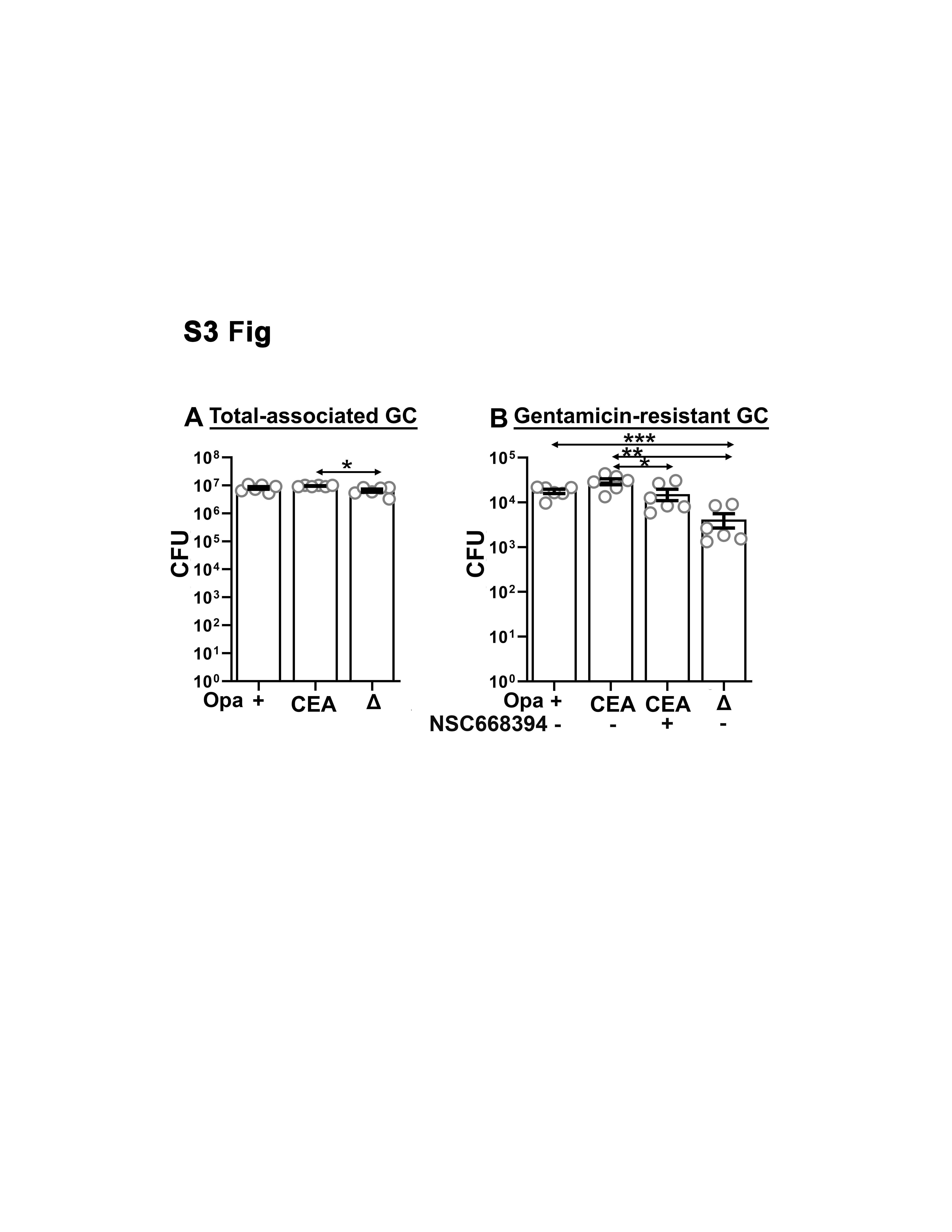

Supplement: S3 Fig — ME-180 were cultured on transwells for 2 days and pretreated with or without the ezrin activation inhibitor NSC668394 (20 μM) for 1 h and inoculated with Pil+Opa+, OpaCEA or ΔOpa GC (MOI~10) from the top for 3 or 6 h with or without the inhibitor. Infected epithelial cells were lysed and cultured before and after gentamicin treatment to determine total epithelial-associated GC (±SEM) at 3 h (A) and gentamicin-resistant GC (±SEM) at 6 h (B). Data points represent individual transwells. n = 2 two independent experiments and three transwells per experiment. *p<0.05; **p< 0.01; ***p<0.001. (TIF) [file ppat.1009592.s003.tif]

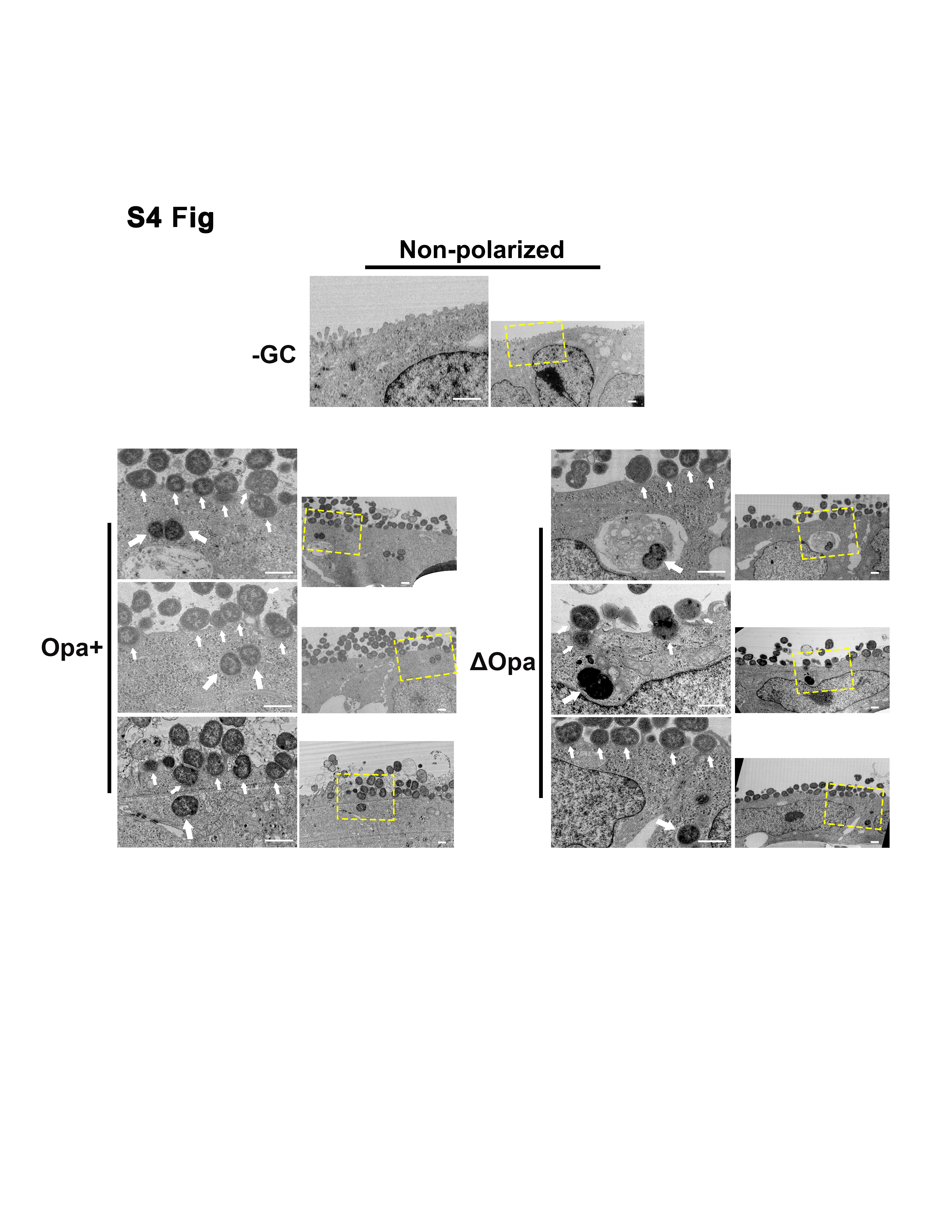

Supplement: S4 Fig — Non-polarized and polarized T84 cells were incubated with Pil+Opa+ or ΔOpa GC (MOI~50) from the top of the chamber for 6 h, fixed, and processed for transmission electron microscopy (TEM). Shown are three sets of images of non-polarized T84 cells with intracellular Opa+ (left) or ΔOpa GC (right). In each set of images, the right panels are original images, and the left panels are enlarged areas within the yellow dash lines. Small arrows, GC directly contacting epithelial membranes. Big arrows, intracellular membrane. (TIF) [file ppat.1009592.s004.tif]

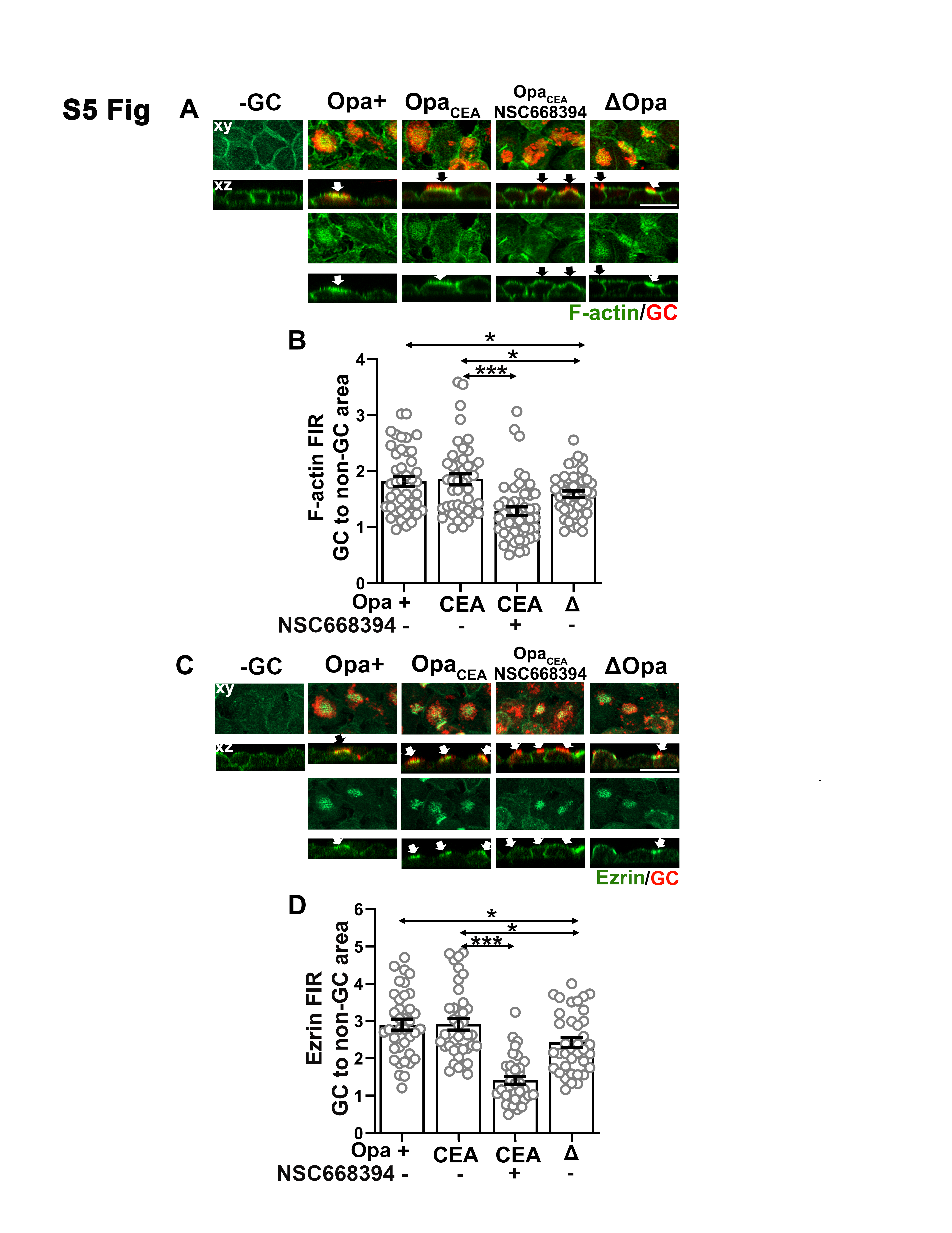

Supplement: S5 Fig — ME-180 were cultured on transwells for 2 days and pretreated with or without the ezrin activation inhibitor NSC668394 (20 μM) for 1 h and inoculated with Pil+Opa+, OpaCEA or ΔOpa GC (MOI~10) from the top of transwells for 6 h with or without the inhibitor. Cells were fixed, stained for F-actin, ezrin, and GC, and analyzed using 3D-CFM. Representative xy images of the top surface and xz images crossing the top and the bottom surfaces are shown (A, C). Arrows indicate the location of GC. The redistribution of F-actin (B) and ezrin (D) was quantified by the mean fluorescence intensity ratio (FIR) (±SEM) of F-actin underneath individual GC microcolonies relative to the adjacent no GC surface area. Data points represent individual GC microcolonies. Scale bar, 20 μm. n = 2 two independent experiments. *p<0.05; ***p<0.001. (TIF) [file ppat.1009592.s005.tif]

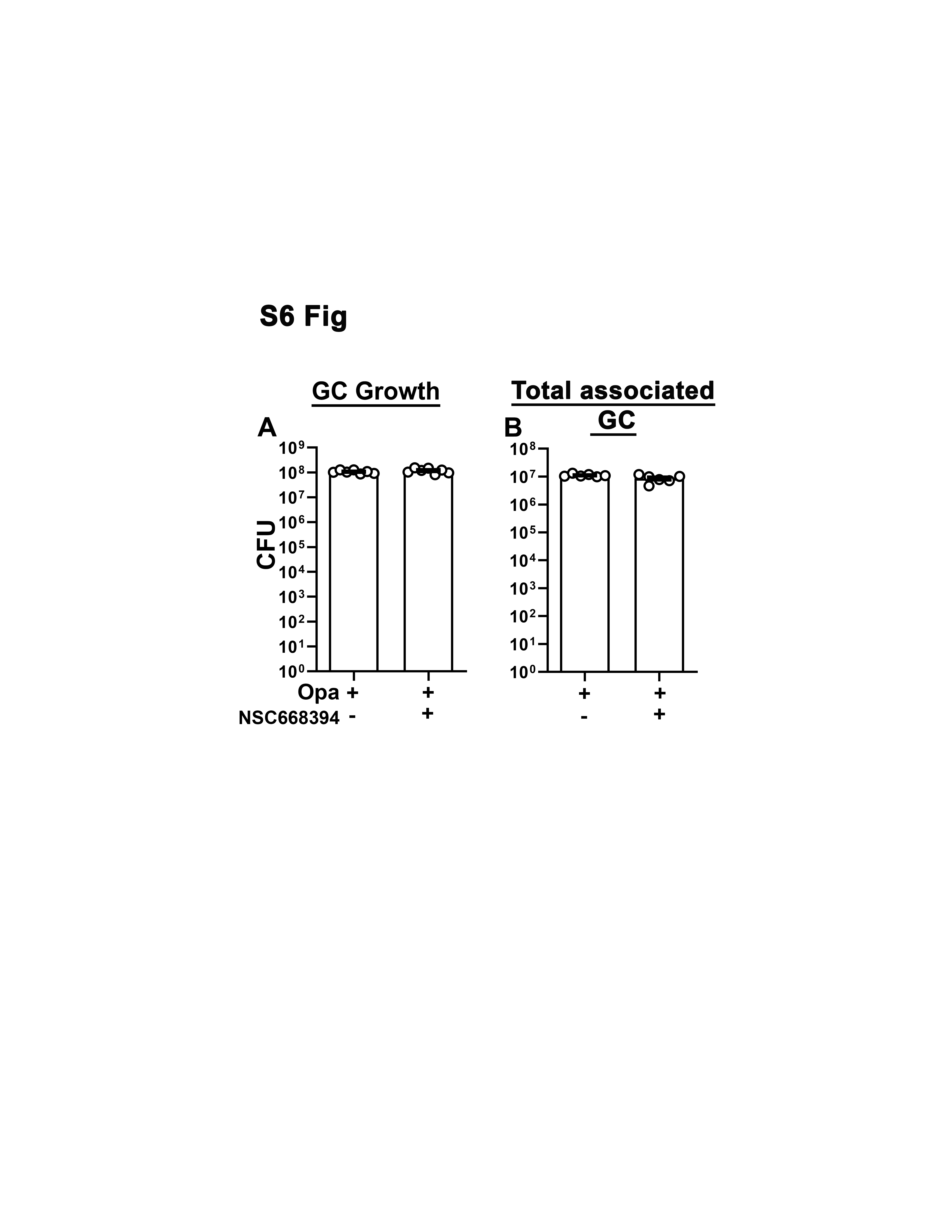

Supplement: S6 Fig — (A) MS11 Pil+Opa+ GC were cultured in DMEM/F12 containing 10% FBS for 6 h in the absence or presence of the ezrin inhibitor NSC668394 (20 μM). CFU was determined by serial dilution and plating on GCK plates. Data points represent individual wells. n = 2 two independent experiments and three wells per experiment. (B) Non-polarized T84 cells were pretreated with or without the ezrin activation inhibitor NSC668394 (20 μM) for 1 h and inoculated with Pil+Opa+ GC (MOI~10) from the top of transwells for 3 h with or without the inhibitor. Total T84-associated GC (±SEM) were quantified by culturing the lysates of infected epithelial cells. n = 2 two independent experiments and three transwells per experiment. (TIF) [file ppat.1009592.s006.tif]

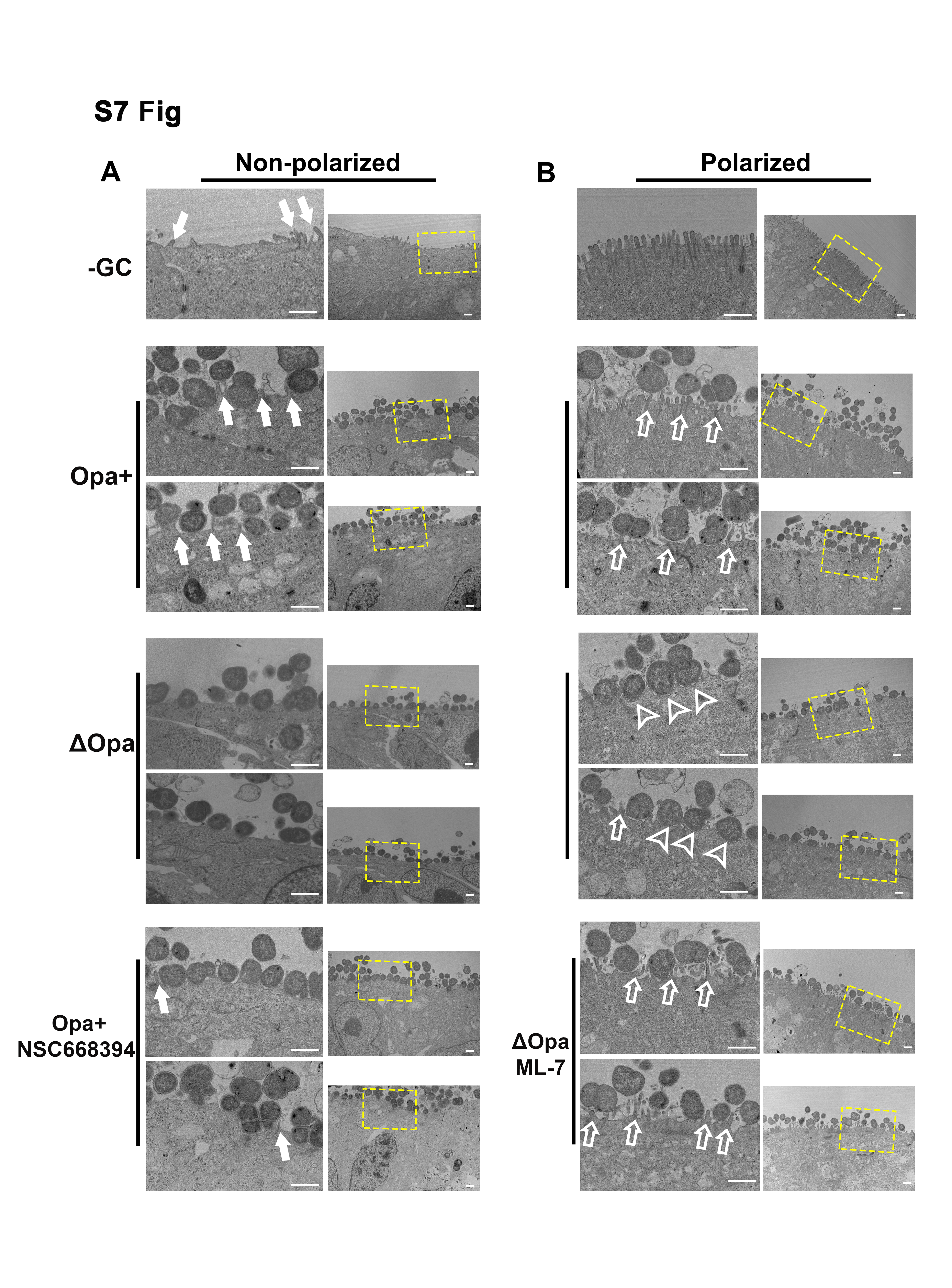

Supplement: S7 Fig — Non-polarized and polarized T84 cells were pre-treated with or without NSC668394 (20 μM) or ML-7 (10 μM) and incubated with Pil+Opa+ or Pil+ΔOpa GC (MOI~50) from the top of transwells for 6 h with or without the inhibitors. Cells were fixed and processed for TEM. Shown are two sets of example images with high (left panels) and low (right panels) magnifications of non-polarized (A) and polarized (B) T84 cells. Yellow dash line rectangles highlight the focused area. Filled arrows, GC-associated elongated microvilli. Open arrows, GC contacting bending microvilli. Open arrowheads, no microvilli visible at GC contact sites. Scale bar, 1 μm. (TIF) [file ppat.1009592.s007.tif]

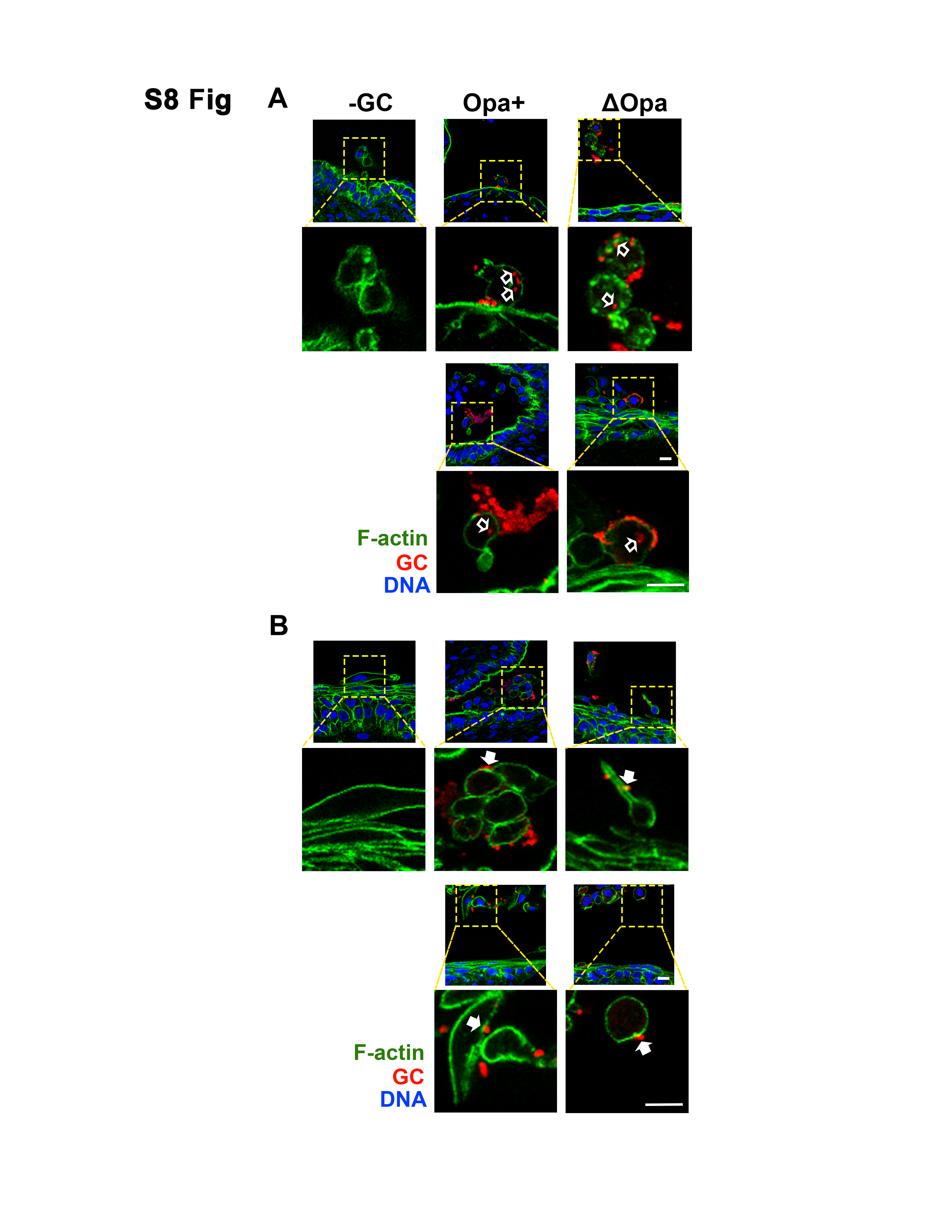

Supplement: S8 Fig — Human cervical tissue explants were incubated with Pil+Opa+ and Pil+ΔOpa (MOI~10) for 24 h, with unassociated GC washed off at 6 and 12 h. Tissue explants were fixed, stained for F-actin, DNA and GC, and analyzed using CFM. Two sets of example images show intracellular GC (A) and F-actin accumulation at GC adherent sites (B) in shedding cervical epithelial cells. Open arrows, intracellular GC. Filled arrows, surface GC microcolonies with F-actin accumulation. Scale bar, 10 μm. (TIF) [file ppat.1009592.s008.tif]
