# Supplementary material for: Gonococcal invasion into epithelial cells depends on both cell polarity and ezrin
Source: PLoS Pathog. 2021 Dec 1;17(12):e1009592. doi: 10.1371/journal.ppat.1009592 (PMC8668114; doi:10.1371/journal.ppat.1009592)
Supplement: Attachment — Submitted filename: Yu et al Point by point PLoS Path.pdf [file ppat.1009592.s009.pdf]

Point-to-point responses to comments

PPATHOGENS-D-21-00895
Gonococcal invasion into epithelial cells depends on both cell polarity and ezrin

We would like to thank the Editors and the Reviewers for your detailed comments and suggestions. These comments are very relevant and helpful. The revision based on the comments has greatly improve the quality of our manuscript. The followings describe how we have addressed comments in the revised manuscript. Modifications have been highlighted in the manuscript.

Editors' comments

1) analyzing more than one time point in binding and invasion

Based on this suggestion, we have compared gonococcal (WT MS11) adherence and invasion in non-polarized and polarized T84 cells on transwells parallelly at three different time points, 3, 6, and 12 h using the gentamicin-resistant assay. We found that at 3 and 6 h, the numbers of gentamicin-resistant bacteria were higher in non-polarized epithelial cells than in polarized epithelial cells, while the total numbers of epithelial-associated bacteria in non-polarized and polarized T84 were similar. By 12 h, the numbers of both epithelial-associated and gentamicin-resistant bacteria in non-polarized and polarized epithelial cells were decreased compared to those at 3 and 6 h, due to bacterial overgrowth, even though unassociated bacteria were washed away at 6 h. This suggests that the effect of the epithelial cell polarity on GC invasion does not depend on time. Therefore, we chose the 6-h for the rest of our study. The new data is included in Fig. 1A-C.

2) using GC constitutively expressing a single Opa in the isogenic delta Opa background for Opa-dependent effects

This is an excellent suggestion. In the revised manuscript, we compared the adherence, invasion of WT MS11 *Neisseria gonorrhoeae* (GC) strain that express phase variable Opa (Opa+) and Opa-deletion strain (ΔOpa) with ΔOpa strain expressing OpaH that binds to Carcinoembryonic antigen-related cell adhesion molecules (CEACAMs) and cannot phase vary (Opa_{CEA}) in non-polarized and polarized T84 cells (Fig. 1D-F). We also compared their impacts on F-actin (Fig. 2A and 2B) and ezrin (Fig. 3A and 3B) distribution. Our results show that Opa_{CEA} GC behave similarly as Opa+ GC, displaying higher numbers of epithelial-associated and gentamicin-resistant bacteria and higher levels of F-actin and ezrin accumulation at GC adherent sites in both non-polarized and polarized epithelial cells than ΔOpa GC. These new results suggest that Opa can enhance GC adherence and invasion and GC-induced F-actin and ezrin accumulation through binding to CEACAMs.

3) considering that exfoliation may occur as a consequence, not cause of invasion

This is an excellent point! In the revised manuscript, we have discussed the possibilities that GC

invasion into cervical epithelial cells induces epithelial shedding, and that GC invasion into cervical epithelial cells after epithelial shedding in the discussion section (Line 602-613).

4) rewriting to frame in light of prior literature on this topic

We have extensively revised the manuscript, particularly the introduction and the discussion sections, to put our data in the context of the extensive early studies on GC invasion into epithelial cells and the underlying mechanism.

5) addressing the discrepancy between gentamicin and TEM data, and addressing the biological significance of the small but statistically significant differences reported

We used both gentamicin resistance and transmission electron microscopy TEM to confirm the effects of cell polarity and Opa expression on GC invasion. Each of the two methods has its advantages and disadvantages. The gentamicin resistance assay is more quantitative and determines the total number of GC surviving the gentamicin treatment in each transwell containing ~$2x10^5$ epithelial cells before inoculation with $2x10^6$ bacteria. However, this method may overestimate GC invasion levels, as gentamicin may not reach bacteria inside microcolonies, particularly Opa+ GC that form big and tight microcolonies [1]. In contrast, TEM allows distinguishing intra- and extra-cellular bacteria visually. However, TEM can only examine a limited number of epithelial cells and GC. Each TEM image only contains two epithelial cells and 10~30 bacteria at a magnification that can distinguish intra- and extra-cellular bacteria rather than all the epithelial cells and bacteria in a transwell. Thus, we have to quantify TEM results using a different method. We apologize for leaving some details in TEM quantification out, and these details have been included in the results (Line 206-219) and methods sections of the revised manuscript. Overall, the results of TEM analysis support the results of the gentamicin resistance assay that epithelial cell polarization inhibits and Opa expression facilitates GC invasion.

We agree with the reviewer's comment. Indeed, the differences in the F-actin FIR between Opa+ GC and ΔOpa GC are small and not obviously visible, even though the differences are significant when a number of GC microcolonies from three independent experiments are quantified. These small differences suggest that Opa proteins can facilitate but are not essential for GC to induce actin reorganization in non-polarized and polarized T84 cells. This finding is consistent with previous reports that Opa proteins are not essential [2, 3] but can facilitate GC invasion into epithelial cells [4-7]. Notably, the differences in the F-actin FIR between GC inoculated non-polarized and polarized T84 cells are much bigger than between Opa+ and ΔOpa GC, suggesting a more substantial role of epithelial cell polarity in regulating GC-induced actin reorganization.

6) using less strong language to describe the results and their interpretation

We greatly appreciate the editors' advise. We have extensively modified the manuscript to describe and interpret our results appropriately in the context of accumulated research in the area.

<u>Reviewers' comments</u>

Reviewer #1:

Part I.

This work examines the differences in the capacity of GC to invade polarized and nonpolarized epithelial cells using a polarizable cell line (T84 cells, intestinal) and cervical explants, which contain the different epithelial cell types within the cervix. Piliated, Opa-positive and piliated Opa-negative gonococci are compared throughout the study. Strengths of the manuscript include i.) the state-of-the art microscopy; ii.) the use of a highly relevant cell culture system (cervical explants) that reproduces the heterogeneity of the histology of the cervix, and iii.) investigation of the mechanism of invasion from the viewpoint of the host cell, specifically whether actin polymerization, ezrin recuritment and phosphorylation differ among these cell types and in response to GC. The mechanistic studies were nicely hypothesis-driven.

Areas where this work could be improved by
i) more discussion of why nonpolarized T84 cells are likely irrelevant system for studying GC invasion, based on what was found with ectocervical cells in cervical explants (which don't have polarity)

Thank you for the suggestion, and we have added a paragraph (Line 615-630) in the discussion section regarding the shortcomings of cell line models, particularly non-polarized epithelia from cell lines, for studying GC invasion in the ectocervix.

ii) adding experimentation with nonpolarized cervical epithelial cells used by many other laboratories to determine whether these are also likely not representing events that occur in whole model systems (i.e. explants) (i.e. it would be very interesting to know whether actin polymerization, ezrin recruitment, etc. occurs in response to GC in ME180 cells in a way that is similar to that of nonpolarized T84 cells).

The revised manuscript provides new data on GC adherence and invasion in ME180 cells cultured on transwells for two days (new supplementary S3 Fig and S5 Fig). Gentamicin resistance assay showed that GC invaded into ME180 cells, and Opa expression facilitated GC invasion (S3 Fig). Immunofluorescence microscopic analysis showed that GC interaction with ME-180 cells induced accumulation of F-actin and ezrin at adherent sites, and Opa expression enhanced the accumulation (S5 Fig). These results are consistent with what we observed in non-polarized T84 cells.

iii) the lack of staining polarized and nonpolarized cells for CEACAMs, which seems important in light of occasional differences between Opa-positive versus Opa-negative bacteria, and the lack of genetic complementation for in experiments in which a difference between Opa-positive

and Opa-negative GC was found.

The revised manuscript provides new data on comparing MS11 strain that expresses a non-phase variable, CEACAM-binding OpaH (Opa_{CEA}) with WT MS11 (Opa+) and its Opa-deletion mutant ΔOpa in new Fig. 1D-F and Fig. 2. Our data show that the expression of Opa_{CEA} enhances GC invasion into non-polarized and polarized T84 cells (Fig. 1D-F) and increases F-actin and ezrin at GC adherence sites (Fig. 2), similar to Opa+ GC that express phase-variable Opa. These results confirm the role of Opa_{CEA} in GC invasion by enhancing actin reorganization.

Furthermore, the authors show the interesting observation that intracellular GC are seen in shed cervical epithelial cells but do not discuss this information in the context of Hauck paper (Muenzner et al J Cell Biol. 2005) which demonstrates the effect of Opa engagement of CEACAMS on epithelial cell shedding. (there are several other papers or reviews by this group that discuss this topic).

This is an excellent point! In the revised manuscript, we discussed the possibilities of GC invading into cervical epithelial cells, inducing epithelial shedding, and GC invading into cervical epithelial cells after epithelial shedding in the discussion section (Line 602-613).

It would also be useful to the reader to place these results, as well as the rationale of the study, in the context of what was previously reported by these authors using the cervical explant system (Yu et al, PLoSPath 2013) so that a larger view of GC invasion through and between cells, with and without CEACAMs could be made. A figure that depicts a model that ties these two pieces of work together would be very useful in summarizing what the cervical explant system has shown.

This is an excellent suggestion. We have included a paragraph (Line 632-661) in the discussion section and a new Fig. 9 to describe our working model on cellular mechanisms by which GC infect the cervical epithelium.

Part II

1. Experiments should be done with nonpolarized cervical cells to determine whether the high rate of invasion of nonpolarized T84 cells and changes in actin, ezrin and phosphorylation are unique to the T84 intestinal cell line, and to confirm this important and interesting observation in a frequently used cervical cell line.

The revised manuscript provides new data on GC adherence and invasion in ME180 cells cultured on transwells for two days (new supplementary S3 Fig and S5 Fig). Gentamicin resistance assay showed that GC invaded into ME180 cells, and Opa expression facilitated GC invasion (S3 Fig). Immunofluorescence microscopic analysis showed that GC interaction with ME-180 cells induced accumulation of F-actin and ezrin at adherent sites, and Opa expression enhanced the accumulation (S5 Fig). These results are consistent with what we observed in non-

polarized T84 cells.

2. A complemented opa-negative mutant should be used for all experiments in which a difference was found for Pil+,Opa+ versus Pil+, Opa- GC. Introduction of a single opa gene should be sufficient for the complemented strain. CEACAM staining should also be incorporated into the immunofluorescence studies.

This is an excellent suggestion. In the revised manuscript, we compared the adherence, invasion of WT MS11 *Neisseria gonorrhoeae* (GC) strain that express phase variable Opa (Opa+) and Opa-deletion strain (ΔOpa) with ΔOpa strain expressing OpaH that binds to Carcinoembryonic antigen-related cell adhesion molecules (CEACAMs) and cannot phase vary (Opa_{CEA}) in non-polarized and polarized T84 cells (Fig. 1D-F). We also compared their impacts on F-actin (Fig. 2A and 2B) and ezrin (Fig. 3A and 3B) distribution. Our results show that Opa_{CEA} GC behave similarly as Opa+ GC, displaying higher numbers of epithelial-associated and gentamicin-resistant bacteria and higher levels of F-actin and ezrin accumulation at GC adherent sites in both non-polarized and polarized epithelial cells than ΔOpa GC. These new results suggest that Opa can enhance GC adherence and invasion and GC-induced F-actin and ezrin accumulation through binding to CEACAMs. We have previously confirmed that Opa_{CEA} expression induces CEACAM recruitment at the surface of T84 cells [8], ectocervical and endocervical epithelial cells [9], by immunofluorescence microscopy.

Part III

The lack of line numbers makes reviewing this manuscript difficult.

Line numbers have been provided in the revised manuscript.

Experimental:
Data pertaining to Fig. 1: A brief description of the details of this experiment with respect to time points should be presented before discussing the data. Adherence was measured at 3 hrs; gentamicin-protected GC were measured at 6 hrs. When was the gentamicin added? While this information is in the methods, it would help the reader understand the data better if the assay is described better here. Similarly, to describe the T84 cells used as cells incubated for 2 days or 10 days in many figure legends is not as informative as describing them as nonpolarized and polarized as was done in the Figure 3 legend.

We have provided a brief description of the experiment for Fig. 1 in the result section (Line 167-172) and the figure legend of the revised manuscript.

Differences in the results when based on the Gm protection assay: While the greater sensitivity of the Gm protection assay compared to microscopy methods is discussed, it is also possible that the Gm protection assay if incomplete exposure of extracellular GC to the Gm due to micro-crevices/folding in the cells or inadequate washing. This finding is also potentially useful to

others who use this well-established assay. The recovery of greater than 1,000 Gm-protected CFU, for example, in Figure 1 corresponds to a value of 16 % or 7% intracellular GC (number of GC/100 epithelial cells). Is this difference in the two assays consistent for all experiments done in this paper?

We agree with the reviewer's comment. We used both gentamicin resistance and transmission electron microscopy TEM to confirm the effects of cell polarity and Opa expression on GC invasion. Each of the two methods has its advantages and disadvantages. The gentamicin resistance assay is more quantitative and determines the total number of GC surviving the gentamicin treatment in each transwell containing ~2x10^5 epithelial cells before inoculation with 2x10^6 bacteria. However, this method may overestimate GC invasion levels, as gentamicin may not reach bacteria inside microcolonies, particularly Opa+ GC that form big and tight microcolonies [1]. In contrast, TEM allows distinguishing intra- and extra-cellular bacteria visually. However, TEM can only examine a limited number of epithelial cells and GC. Each TEM image only contains two epithelial cells and 10~30 bacteria at a magnification that can distinguish intra- and extra-cellular bacteria rather than all the epithelial cells and bacteria in a transwell. Thus, we have to quantify TEM results using a different method. We apologize for leaving some details in TEM quantification out, and these details have been included in the results (Line 207-219) and methods sections of the revised manuscript. Overall, the results of TEM analysis support the results of the gentamicin resistance assay that epithelial cell polarization inhibits and Opa expression facilitates GC invasion.

Occasionally there is a difference when using Opa-positive versus Opa-negative Gc, for example in the enrichment of ezrin in infected nonpolarized cells and the detection of intracellular GC in endocervical and ectocervical cells (Fig. 5A). A complemented Opa-negative strain (i.e. with one opa gene) should be used to confirm these differences are due to lack of Opa expression.

This is an excellent suggestion. In the revised manuscript, we compared the adherence, invasion of WT MS11 *Neisseria gonorrhoeae* (GC) strain that express phase variable Opa (Opa+) and Opa-deletion strain (ΔOpa) with ΔOpa strain expressing OpaH that binds to Carcinoembryonic antigen-related cell adhesion molecules (CEACAMs) and cannot phase vary (Opa_{CEA}) in non-polarized and polarized T84 cells (Fig. 1D-F). We also compared their impacts on F-actin (Fig. 2) and ezrin (Fig. 3A and 3B) distribution. Our results show that Opa_{CEA} GC behave similarly as Opa+ GC, displaying higher numbers of epithelial-associated and gentamicin-resistant bacteria and higher levels of F-actin and ezrin accumulation at GC adherent sites in both non-polarized and polarized epithelial cells than ΔOpa GC. These new results suggest that Opa can enhance GC adherence and invasion and GC-induced F-actin and ezrin accumulation through binding to CEACAMs.

With respect to differences in results when Opa-positive GC are used, CEACAM staining should be performed on polarized and nonpolarized T84 cells to confirm that Opa interactions with these receptors is occurring (or not), and to see if the CEACAMs are on the apical side of polarized cells or localized (i.e. recruited) differently when GC are present.

As discussed above, the new data provided in the revised manuscript (Fig. 1D-G, Fig. 2, and Fig. 3A and 3B) showed that MS11 GC expressing phase variable Opa proteins (Opa+) behave

similarly to MS11 GC expressing non-phase variable, CEACAM-binding OpaH (Opa_{CEA}) in regulating GC adherence and invasion, as well as actin reorganization, in non-polarized and polarized T84 cells. These data suggest that most Opa proteins expressing on Opa+ GC are CEACAM-binding. Our previous studies (Yu et al. 2019. PLoS Path) [8, 9] have shown that Opa_{CEA} GC colonization recruits CEACAMs to adherent sites by immunofluorescence.

There are many studies on GC invasion of nonpolarized ME180 cells and primary cervical cells. This should be discussed with respect to the potential lack of polarity in these specific cell systems and how that may influence actin and ezrin recruitment and localization. Could it be that GC is not as invasive as is thought, based on these nonpolarized cell models? Also, no mention is made of the well-defined invasion pathway discovered by Jennifer Edwards, which leaves the discussion incomplete for the reader who is trying to put this all together.

We have modified the discussion section extensively in the revised manuscript to put our findings in the context of the accumulated studies in the last four decades. We have included the studies from Dr. Jennifer Edwards' group in the discussion and suggested that the binding of pili to CR3 is a potential way to activate ezrin and actin reorganization for GC invasion. Even though we still lack direct evidence, our data from human cervical tissue explants suggest lower invasiveness of GC (GC entry into epithelial cells) *in vivo* than *in vitro* during the first 24 h window. Low ezrin expression and epithelial cell polarity are likely two of the factors that contribute to this low GC invasiveness, even though CR3 is expressed.

The cervical explant system used by the authors is a unique and powerful system in its relevance and capacity to compare differences in different anatomical sites within the cervix. It is puzzling to this reviewer why they do not build on their previous work (PLosPath 2015) with this system to better explain what they are testing and why (in the introduction) and to provide the reviewer with a larger and more comprehensive summary of what has been learned with this system in the discussion.

This is an excellent suggestion. We have included a paragraph (Line 632-661) in the discussion section and a new Fig. 9 to describe our working model on cellular mechanisms by which GC infect the cervical epithelium.

Shedding should be discussed in the context of C. Hauck's work in this area.

This is an excellent point! In the revised manuscript, we discussed both possibilities of GC invading into cervical epithelial cells, inducing epithelial shedding, and GC invading into cervical epithelial cells after epithelial shedding in the discussion section (Line 602-613)

Minor:
Last sentence of top paragraph, page 10, is incomplete or missing some words.

Corrected accordingly.

The opening line of the abstract states that GC causes symptomatic infections in the cervix but in the introduction, more emphasis is placed on the high percentage of asymptomatic infections in

women. This is confusing and because symptomology doesn't have much to do with this paper, this could be removed from the abstract.

Thanks for the suggestion. We have made the correction accordingly.

Reviewer #2

Part I

In this study, Yu et al. employ two in vitro models (immortalized T84 cells, and cervical explants) to analyze the mechanisms of gonococcal invasion in cells with various morphologies. Building on prior work from their group and others, they present descriptive results on cell morphology during infection, as well as perturbation experiments which suggest roles for actin, the actin-membrane linker ezrin, and signaling events including ezrin phosphorylation and Ca^{2+} signaling in host cell responses and bacterial invasion.

In general the experiments are well-controlled and described and the experimentation is extensive. The major experimental limitations (see below) are the use of pharmacological inhibitors that may have off-target effects, and a focus on analyses conducted at only a single time point (in most cases, 6 h post-infection). Most of the microscopy is beautiful.

We thank the reviewer for appreciating our microscopic work and pointing out the limitation of our pharmacological approach and single time point examination. In the revised manuscript, we have discussed the possible off-target effects of inhibitors. Because the molecules we focused on are essential for various functions of epithelial cells, the gene knockdown and knockout approach would have broad impacts on T84 cells, particularly viability and polarity. To minimize the off-target effects of inhibitors used, we selected minimal effective concentrations. We also tested the effects of inhibitors on GC growth and adherence (for the ezrin inhibitor NSC668394, please see S6 Fig in the revised manuscript and for the non-muscle myosin II light chain kinase inhibitor ML-7 and the Ca^{2+} flux inhibitor 2APB, please see our previous publications [9, 10]).

Based on reviewer's suggestions, we have compared gonococcal (WT MS11) adherence and invasion in non-polarized and polarized T84 cells on transwells parallelly at three different time points, 3, 6, and 12 h using the gentamicin-resistant assay. We found that at 3 and 6 h, the numbers of gentamicin-resistant bacteria were higher in non-polarized epithelial cells than in polarized epithelial cells, while the total numbers of epithelial-associated bacteria in non-polarized and polarized T84 were similar. By 12 h, the numbers of both epithelial-associated and gentamicin-resistant bacteria in non-polarized and polarized epithelial cells were decreased compared to those at 3 and 6 h, due to bacterial overgrowth, even though unassociated bacteria were washed away at 6 h. This suggests that the effect of the epithelial cell polarity on GC invasion does not depend on time. Therefore, we chose the 6-h for the rest of our study. The new data is included in Fig. 1A-C.

The manuscript itself raises more significant concerns. As mentioned above, the experiments build on older work. Key papers, sometimes with nearly identical experiments, are not cited as prior art, and in some cases the data presented appear at odds with older results. It will be essential in any resubmission to address these issues. In particular, divergence between the present manuscript and other published data should be highlighted so that inconsistencies can be addressed (and, one hopes, resolved) in future work.

This is an excellent point! We have extensively revised the manuscript to put this study in the contest of accumulated research on GC invasion into epithelial cells over the four decades. In the introduction section, we expand our literature review to include critical findings of the previous literature. In the discussion section, we expand the discussion on the similarity and differences between previous studies and this study and future directions.

Part II

Most of the following issues do NOT require additional experimentation, but are still important. It might be possible, with extensive rewriting, or presentation of control data collected but not presented, to address the following concerns without additional experimentation.

1. The authors seem to imply (Results, first paragraph) that the choice of the T84 model, or its use in polarized and non-polarized configurations, is novel. It is not. The first use of this system to study both N. meningitidis and N. gonorrhoeae infection was published in 1996 (Merz et al., PMID: 8972489). Reassuringly, the adhesion, invasion, and (for polarized cells) transepithelial migration data presented here are very close to the data that we reported for both polarized and non-polarized T84 cells 25 years ago. Many of the T84 results are also consistent with data published soon thereafter by others who are *also* not cited (Criss et al., PMID: 16922862; Pujol et al., Wang et al., PMID: 17683982; Pujol et al., PMID: 9353073). These papers need to be cited.

We agree with the reviewer that non-polarized and polarized T84 cells have been used for studying GC invasion since 1996. The reviewer established these models. That is one of the reasons for us to choose T84 cells as our model. Our data that Opa expression enhances GC invasion in both non-polarized and polarized T84 cells are consistent with the early findings, suggesting the T84 cell model work appropriately. We have extensively revised the manuscript to put our data in the contest of previous studies, including the references suggested by the reviewer. Different from previous studies, we set up both non-polarized and polarized T84 on transwells with the same number of T84 cells per transwells and parallelly compared GC adherence, invasion, and induced actin reorganization in non-polarized and polarized epithelial cells. This enables us to reveal the distinctive mechanisms by which GC interact and invade into non-polarized and polarized epithelial cells. We have clarified this point in the revised manuscript.

1a. Similarly, on p. 22, the authors write: "Our findings that Opa expression promotes GC invasion into T84 epithelial cells, no matter if they are non-polarized and polarized using

gentamicin resistant assay, are consistent with previous reports [11] [45]." But that paper (Grassmé) does not employ T84 or any other polarized cell line. It uses Chang cells, now known to be a HeLa derivative.

We apologize for our wrong wording. We intended to state that our results are consistent all previous reports on the enhancing role of Opa on GC invasion into various epithelial cells, including T84. In the revised manuscript, we have modified the sentence to correctly reflect our points.

1b. In Fig. 1, why are the invasion indices (ratio of invasion : cell association) so much higher when assessed by TEM vs. Gm protection assay?

TEM can only examine a limited number of epithelial cells and GC. Each TEM image only contains two epithelial cells and 10~30 bacteria and often cannot cover an entire bacterial microcolony at a magnification that can distinguish intra- and extra-cellular bacteria. Thus, we have to quantify TEM results using a different method from the gentamicin-resistant assay. Eight images were randomly acquired from each condition and each of three independent experiments. We found that ~25% of the images from GC-inoculated non-polarized T84 cells but none of the eight images from GC-inoculated polarized T84 cells showed intracellular GC. We quantified the percentage of intracellular GC over the total number of GC directly contacting epithelial cells in individual images using the images showing intracellular GC. We observed ~16% Opa+ and ~7% ΔOpa GC inside non-polarized T84 cells (Fig. 1H). In the new Fig. 1G, we add big arrows to indicate intracellular GC and small arrows to indicate GC directly contacting the plasma membrane of epithelial cells. We apologize for leaving some details in TEM quantification out previously, and these details have been included in the results (Line 207-219) and methods sections of the revised manuscript. Overall, the results of TEM analysis support the results of the gentamicin resistance assay that epithelial cell polarization inhibits and Opa expression facilitates GC invasion.

2. The authors say (P. 12, 1st paragraph) that Ca^{2+} signaling does not occur with non-polarized cells (Fig. 3). That claim appears to be in direct contradiction to prior results (again, key work by other authors is not cited — e.g., Källström et al., PMID: 9705315; Ayala et al., PMID: 11298650 and PMID: 16309460; Müller et al., PMID: 9889191). However, the earlier experiments demonstrated Ca^{2+} signaling on a time scale of minutes, while the present manuscript apparently assays elevated Ca^{2+} only at a 4h time point.

2a. The prior art needs to be cited and discrepancies between those studies and the present work need to at least be mentioned and preferably discussed.

We agree with the reviewer's comment. The revised manuscript has provided additional discussion on the early Ca^{2+} flux minutes post GC inoculation in epithelial cells shown by included the suggested references and the elevated cytoplasmic level of Ca^{2+} at 4 h in GC-inoculated polarized T84 (Line 567-573).

2b. Given previously reported results, what might have happened during the previous 4h?

This is an interesting question that requires further investigation. We have expanded the discussion section to discuss this question in revised manuscript (Line 567-573). Early studies have shown that GC interaction can trigger a transient Ca^{2+} flux in non-polarized epithelial cells minutes post-inoculation [12-14]. The relationship between GC-induced Ca^{2+} flux at the early time and the elevated cytoplasmic Ca^{2+} level hours later is unclear. If the early Ca^{2+} influx does happen in the polarized T84 cells, the persistent Ca^{2+} influx could be one possibility for the elevated cytoplasmic Ca^{2+} level 4-h post GC inoculation. Another possibility is that the later Ca^{2+} elevation is induced by a different mechanism. We have previously shown that the elevated cytoplasmic Ca^{2+} in polarized T84 cells observed at later times is sensitive to 2APB, an inhibitor inhibiting Ca^{2+} release from the intracellular storage, suggesting an involvement of GC-induced signaling. However, the relationship between the early and late Ca^{2+} flux induced by GC is beyond the scope of this manuscript.

2c. Minor point: the authors say that Fluo-4 is used to detect Ca^{2+}, but Fluo-4 is cell-impermeant. If an AM ester was used that should be specified and the product number noted.
Correct the name.

We have made the correction in the revised manuscript.

3. The descriptions of the roles of microvilli are very interesting, but could be considerably clarified and sharpened. For example, it's pretty clear that it's microvillus *remodeling or dynamics*, not the presence or absence of microvilli per se, that controls the ability of the pathogen to invade cells. That's clearly stated in the Discussion (top of p. 20), but much less clear elsewhere in the manuscript.

We have modified wording accordingly to clarify this point.

4. The general conclusion that polarity, per se, (and especially cytoskeleton polarity) is controlling GC entry, is found throughout the manuscript, but is not particularly well-supported. The development of polarity is accompanied by other changes that occur during differentiation, such as the sequestration of receptors on the basolateral surface or differences in expression of relevant receptor or downstream signaling molecules. Modulating polarity by simply changing the time after plating the cells onto transwell filters does not control for these potential differences. This limitation needs to be discussed.

We agree with the reviewer's comments and discussed the limitation of the cell line model in the revised manuscript (Line 615-630). Our previous studies and this study have partially characterized polarized T84 cells cultured on transwells. We have shown that T84 cells form the tight junction and the adherens junction at the apical surface (S1 Fig, S2 Fig) [10] and polarize the distribution of ezrin-actin networks (Fig. 2, Fig. 3, and S2 Fig), actomyosin [10], and the Opa host receptors CEACAMs apically [8], and epidermal growth factor receptor basolaterally [15].

However, T84 cells cultured on transwells may not have the same transcriptional and protein expression profiles and polarized distributing molecules exactly as endocervical epithelial cells.

5. The small molecule inhibitors used, particularly the ezrin inhibitor, are not particularly well-characterized. 2-APB is known to have fairly complex effects and to differentially target different classes of Ca2+ channels, even acting as an *agonsist* of Trpv channels at concentrations similar to those used here (Gao, PMID: 26876731) Again, the experimental limitation should at least be mentioned, with appropriate pointers to literature.

The revised manuscript has discussed the limitation of the inhibitor approaches and included references regarding their off-target effects.

6. The experiments with cervical explants are particularly useful, and represent a real step forward. However, there are again some unstated limitations.

6a. First and foremost, there is the curious result that little or no invasion was detected in endocervical or ectocervical cells. Instead, bacteria were noted in shed cells, leading to the authors' conclusion that GC may prefer to enter shedding cells. The alternative conclusion, of course, is that GC entry *causes* the cells to delaminate. Both alternative hypotheses should be mentioned.

This is an excellent point! In the revised manuscript, we discussed the possibilities of GC invading into cervical epithelial cells, inducing epithelial shedding, and GC invading into cervical epithelial cells after epithelial shedding in the discussion section (Line 602-613).

6b. The lack of invasion noted in explants could conceivably reflect not just terminal differentiation or cornification, but cell death. Are the cells that are not invaded alive? Was viability examined?

Cornification of epidermal keratinocytes is a terminal differentiation process and a programmed cell death, which leads to the formation of the outermost skin barrier [16]. It is unknown whether the ectocervical epithelium undergoes a similar process, but it is one of the possibilities. Unfortunately, it is not technically possible yet to determine the viability of epithelial cells in cervical tissues. However, it is definitely one of our future interests.

7. As mentioned above, the study's biggest limitation, to my mind, is the examination of single time points rather than trajectories. The choices of time points need to be explained, and the limitations of using single time points (we can't see what we don't look at) need to be forthrightly discussed.

Based on this suggestion, we have compared gonococcal (WT MS11) adherence and invasion in non-polarized and polarized T84 cells on transwells parallelly at three different time points, 3, 6,

and 12 h using the gentamicin-resistant assay. We found that at 3 and 6 h, the numbers of gentamicin-resistant bacteria were higher in non-polarized epithelial cells than in polarized epithelial cells, while the total numbers of epithelial-associated bacteria in non-polarized and polarized T84 were similar. By 12 h, the numbers of both epithelial-associated and gentamicin-resistant bacteria in non-polarized and polarized epithelial cells were decreased compared to those at 3 and 6 h, due to bacterial overgrowth, even though unassociated bacteria were washed away at 6 h. This suggests that the effect of the epithelial cell polarity on GC invasion does not depend on time. Therefore, we chose the 6-h for the rest of our study. The new data is included in Fig. 1A-C.

Reviewer #3

Part I

Yu et al. examine use microscopy to examine the roles of cell polarization, ezrin, and F-actin in gonococcal interactions with human cells. An invasion method is described that occurred in non-polarized T84 colonic cells and in cervical cells found in the medium of cervical explant cultures, though the mechanisms leading to cell infection and exfoliation were not determined. By contrast, invasion was said not to occur in polarized T84 cells or in cervical explant tissue, raising questions about the significance of this invasion phenomenon.

Part II

1. The biological importance of the differences in FIR reported throughout the paper are difficult to discern, particularly when the differences are so small. For instance, in Fig. 1F the authors report a statistically-significant difference in F-actin FIR between Opa+ GC infections and delta-opa GC infections of non-polarized cells, and yet the averages appear to be 1.4 and 1.2, respectively, and the points in the delta-opa infection condition appear to completely overlap with those in the Opa+ condition, with only eight points in the Opa+ condition outside this range. Are these results actually different? Certainly the micrographs provided (Fig 1E) do not show any discernable difference between the Opa+ and delta-opa infections.

We agree with the reviewer's comment. Indeed, the differences in the F-actin FIR between Opa+ GC and ΔOpa GC are small and not obviously visible, even though the differences are significant when 54~81 GC microcolonies from three independent experiments are quantified. These small differences suggest that Opa proteins can facilitate but are not essential for GC to induce actin reorganization in non-polarized and polarized T84 cells. This finding is consistent with previous reports that Opa proteins are not essential [2, 3] but can facilitate GC invasion into epithelial cells [4-7]. Notably, the differences in the F-actin FIR between GC inoculated non-polarized and polarized T84 cells are much bigger than between Opa+ and ΔOpa GC, suggesting a more substantial role of epithelial cell polarity in regulating GC-induced actin reorganization.

2. Fig. 1 claims to report both invasion (1B) and intracellular GC (1D), yet these two

experiments yield different results. The authors conclude from 1D that no intracellular GC are present in polarized cells, while the standard invasion assay in 1B shows maybe 30% as many GC in the polarized cells as in the non-polarized.

We used both gentamicin resistance and transmission electron microscopy TEM to confirm the effects of cell polarity and Opa expression on GC invasion. Each of the two methods has its advantages and disadvantages. The gentamicin resistance assay is more quantitative and determines the total number of GC surviving the gentamicin treatment in each transwell containing ~2x10^5 epithelial cells before inoculation with 2x10^6 bacteria. However, this method may overestimate GC invasion levels, as gentamicin may not reach bacteria inside microcolonies, particularly Opa+ GC that form big and tight microcolonies [1]. In contrast, TEM allows distinguishing intra- and extra-cellular bacteria visually. However, TEM can only examine a limited number of epithelial cells and GC. Each TEM image only contains two epithelial cells and 10~30 bacteria at a magnification that can distinguish intra- and extra-cellular bacteria rather than all the epithelial cells and bacteria in a transwell. Thus, we have to quantify TEM results using a different method. We apologize for leaving some details in TEM quantification out, and these details have been included in the results (Line 207-219) and methods sections of the revised manuscript. Overall, the results of TEM analysis support the results of the gentamicin resistance assay that epithelial cell polarization inhibits and Opa expression facilitates GC invasion.

3. As in Fig 1, the results for Opa+ and delta-opa in Fig 2B and 2G appear very small. The error bars presented do not appear to represent the large spread in the data as represented by the data points shown.

The differences in the ezrin FIR between Opa+ and DOpa GC are consistent with the differences in the F-actin FIR between Opa+ and DOpa GC. As detailed above, these results suggest that Opa proteins can facilitate but are not essential for GC-induced actin reorganization in non-polarized and polarized T84 cultured on transwells, as discussed above. Data points represent individual microcolonies. The statistical analysis is based on 54~81 individual GC microcolonies from three independent experiments, and error bars are based on the standard error of the mean (SEM) for a large number of data points.

4. It is unclear if the microscopy experiments were repeated, i.e., does the “n=3” found in the figure legends mean that the experiment was done on three different days with three different cultures, or does it mean that it was done once with three technical replicates?

We performed the experiment on three different days with three different culture. We have changed n=3 to three independent experiments in the revised manuscript.

5. One of the most interesting results of the study is the observation of sloughed cervical cells with internalized gonococci. The authors claim both that quantification of this process is impossible and that the number of gonococci that invaded cervical cells is less than the number

that remain on the surface. Isn't it possible that gonococci invaded significant numbers of cervical cells that were then sloughed from the epithelium? They should collect sloughed cells from the medium and plate the bacteria, add more earlier time points to the microscopy studies to try to see the invasion and sloughing process, and quantify cell sloughing as has been done by Muenzner (Science 329:1197).

We thank the reviewer for appreciating our results and for the excellent suggestion. Indeed, we can estimate the relative number of epithelial cells shed from the cervical tissue (see our previous publications [9, 10]), similar to Dr. Hauck's group did with a mouse model [17]. To quantify the number of exfoliated epithelial cells with intracellular GC, we did try to harvest exfoliated epithelial cells from culture supernatants before our initial submission. However, we got very low numbers of epithelial cells, suggesting that most exfoliated epithelial cells were not in the culture supernatant. We suspect that most exfoliated epithelial cells may remain loose association with the cervical tissue and may be removed during washing, and some of them may attach to the surface of the culture well surface [18], which made quantification almost impossible. Importantly, even if we could collect epithelial cells from all these factions, we still cannot distinguish whether GC enter epithelial cells before or after epithelial cells shed off. Limited availability of human cervical tissues prevents us from analyzing multiple time points. To address these issues, we are currently developing live imaging of cervical tissue using the multiphoton intravital microscopic technique, but this is beyond the scope of this manuscript.

While we currently cannot experimentally address this question, we revised the discussion section to include the possibility of GC invasion before epithelial exfoliation, causing epithelial shedding.

6. The authors' previous cervical tissue studies identified the transition zone as the region of the cervix where gonococci invade tissue (Yu et al. doi.org/10.1371/journal.ppat.1008136). Since the authors in that study reported non-polarized cells in the transition zone, it seems quite possible that the invasion phenomenon studied in the current manuscript could be going on in those cells. Why was the transition zone not examined in this study? Thus the conclusions in the last paragraph of the discussion that the cervix is protected from GC invasion may be wrong.

We did not include transformational zone because epithelial cells in this location are extremely heterogeneous, gradually increasing cell polarity from the ectocervical side into the endocervical side. The area of the transformational zone also different from one cervix to the other. We do not have a cell line model to mimic such a polarity gradient. In addition, transformational zone epithelial cells express a low level of CEACAMs. While GC invasion in the transformational zone is very interesting and important, its complexity requires sole focus that is beyond the scope of the current manuscript. We agree with the reviewer's comments and revised this part of the discussion accordingly. The low expression of ezrin and the epithelial cell polarization may protect the ectocervix and the endocervix from GC invasion, respectively.

7. The authors should explain why their results with T84 cells are different from those of Merz and So, particularly with regard to invasion and transcytosis (doi:10.1146/annurev.cellbio.16.1.423.).

The revised manuscript has extensively revised the introduction and the discussion section to put our study in the context of the research accumulated in the last four decades.

The data presented in this manuscript is largely consistent with the studies reviewed and published by Merz and So [6, 19, 20] on GC invasion and Opa's roles in GC invasion. GC entering epithelial cells from the apical surface can exit from the basolateral surface, leading to transcytosis. We have recently shown that GC transmigration across polarized epithelial cells, including endocervical epithelial cells, is associated with their ability to disrupt the apical junction that seals the paracellular space [9, 10, 21]. The lower level of intraepithelial GC than subepithelial GC after 24 h incubation supports that GC can transmigrate across the epithelium by breaking the apical junction, but does not exclude the transcytosis pathway, such as GC entering and exiting epithelial cells rapidly or GC entery and transcytosis of the epithelium after 24 h. Because GC transmigration across the epithelium is not the focus of this manuscript, we have decided not to discuss this point.

Part III

1. The use of red and green for microscopy images is not optimal for color-blind readers.

We sincerely apologize to color-blind readers but do not have a better choice. We chose the green and red combination because it displays the colocalization best (yellow).

2. The addition of more micrographs (added to the supplement) or larger micrographs might better support the study.

Based on the reviewer's suggestion, the revised manuscript has provided more samples of TEM images in new S4 Fig and S7 Fig.

3. Methodological details are missing. What is the anti-gonococcal antibody used? What method was used for permeabilization of cells?

We have revised the methods section to provide the information.

4. The labeling is wrong in Fig 1D (assuming the text correctly describes the results).

We apologize for our mistake and has corrected it in the revised manuscript.

5. P3, line 2. Note that gonorrhea is the second most common reportable STI, not the second most common STI.

Corrected.

6. P4, line 11. "disassembly"

Corrected.

7. P6, line 16. Which CEACAMs are on T84s?

We do not know which isoforms of CEACAMs are expressed by T84 cells. Our previously published study [8] showed that T84 cells were positively stained with an anti-human CEACAM antibody that binds to CEACAM 1, 3, and CEACAM staining was concentrated at the apical surface of polarized T84 cells. Opa_{CEA} but not ΔOpa GC induce the recruitment of CEACAMs. We will cite this reference in the revised manuscript

8. P10, line 7. Awkward, reword.

We have rewritten this sentence.

9. Fig 3F label. F-actin

Corrected.

10. P15, line 3. Only one strain of GC was used in the study. Reword to clarify to WT and mutant.

Corrected.

11. P18, last line. "Prefer" is too much anthropomorphizing. Reword.

Reworded.

12. Include some figure references in the discussion to lead the reader to the points being made.

We have cited figures in the discussion section of the revised manuscript.

13. P20, line 4. "Pili have".

Corrected.

References

1. Wang LC, Litwin M, Sahiholnasab Z, Song W, Stein DC. Aggregation Reduces Its Ceftriaxone Susceptibility. Antibiotics (Basel). 2018;7(2). Epub 2018/06/15. doi: 10.3390/antibiotics7020048. PubMed PMID: 29914058; PubMed Central PMCID: PMCPMC6022932.
2. Song W, Ma L, Chen R, Stein DC. Role of lipooligosaccharide in Opa-independent invasion of Neisseria gonorrhoeae into human epithelial cells. J Exp Med. 2000;191(6):949-60. doi: 10.1084/jem.191.6.949. PubMed PMID: 10727457; PubMed Central PMCID: PMCPMC2193109.
3. Swanson KV, Jarvis GA, Brooks GF, Barham BJ, Cooper MD, Griffiss JM. CEACAM is not necessary for Neisseria gonorrhoeae to adhere to and invade female genital epithelial cells. Cell Microbiol. 2001;3(10):681-91. PubMed PMID: 11580753.
4. Makino S, van Putten JP, Meyer TF. Phase variation of the opacity outer membrane protein controls invasion by Neisseria gonorrhoeae into human epithelial cells. EMBO J. 1991;10(6):1307-15. PubMed PMID: 1673923; PubMed Central PMCID: PMCPMC452788.
5. Kupsch EM, Knepper B, Kuroki T, Heuer I, Meyer TF. Variable opacity (Opa) outer membrane proteins account for the cell tropisms displayed by Neisseria gonorrhoeae for human leukocytes and epithelial cells. EMBO J. 1993;12(2):641-50. PubMed PMID: 8440254; PubMed Central PMCID: PMCPMC413248.
6. Waldbeser LS, Ajioka RS, Merz AJ, Puaoi D, Lin L, Thomas M, et al. The opaH locus of Neisseria gonorrhoeae MS11A is involved in epithelial cell invasion. Mol Microbiol. 1994;13(5):919-28. PubMed PMID: 7815949.

7. Hauck CR, Meyer TF. 'Small' talk: Opa proteins as mediators of Neisseria-host-cell communication. Curr Opin Microbiol. 2003;6(1):43-9. PubMed PMID: 12615218.
8. Stein DC, LeVan A, Hardy B, Wang LC, Zimmerman L, Song W. Expression of Opacity Proteins Interferes with the Transmigration of Neisseria gonorrhoeae across Polarized Epithelial Cells. PLoS One. 2015;10(8):e0134342. Epub 2015/08/05. doi: 10.1371/journal.pone.0134342. PubMed PMID: 26244560; PubMed Central PMCID: PMCPMC4526573.
9. Yu Q, Wang LC, Di Benigno S, Gray-Owen SD, Stein DC, Song W. Neisseria gonorrhoeae infects the heterogeneous epithelia of the human cervix using distinct mechanisms. PLoS Pathog. 2019;15(12):e1008136. Epub 2019/12/02. doi: 10.1371/journal.ppat.1008136. PubMed PMID: 31790511; PubMed Central PMCID: PMCPMC6907876.
10. Wang LC, Yu Q, Edwards V, Lin B, Qiu J, Turner JR, et al. Neisseria gonorrhoeae infects the human endocervix by activating non-muscle myosin II-mediated epithelial exfoliation. PLoS Pathog. 2017;13(4):e1006269. Epub 2017/04/13. doi: 10.1371/journal.ppat.1006269. PubMed PMID: 28406994; PubMed Central PMCID: PMCPMC5391109.
11. Wang J, Gray-Owen SD, Knorre A, Meyer TF, Dehio C. Opa binding to cellular CD66 receptors mediates the transcellular traversal of Neisseria gonorrhoeae across polarized T84 epithelial cell monolayers. Mol Microbiol. 1998;30(3):657-71. PubMed PMID: 9822830.
12. Källström H, Islam MS, Berggren PO, Jonsson AB. Cell signaling by the type IV pili of pathogenic Neisseria. J Biol Chem. 1998;273(34):21777-82. doi: 10.1074/jbc.273.34.21777. PubMed PMID: 9705315.
13. Ayala BP, Vasquez B, Clary S, Tainer JA, Rodland K, So M. The pilus-induced Ca2+ flux triggers lysosome exocytosis and increases the amount of Lamp1 accessible to Neisseria

IgA1 protease. Cell Microbiol. 2001;3(4):265-75. doi: 10.1046/j.1462-5822.2001.00112.x. PubMed PMID: 11298650.

14. Ayala P, Wilbur JS, Wetzler LM, Tainer JA, Snyder A, So M. The pilus and porin of Neisseria gonorrhoeae cooperatively induce Ca(2+) transients in infected epithelial cells. Cell Microbiol. 2005;7(12):1736-48. doi: 10.1111/j.1462-5822.2005.00586.x. PubMed PMID: 16309460.
15. Swanson KV, Griffiss JM, Edwards VL, Stein DC, Song W. Neisseria gonorrhoeae-induced transactivation of EGFR enhances gonococcal invasion. Cell Microbiol. 2011;13(7):1078-90. doi: 10.1111/j.1462-5822.2011.01603.x. PubMed PMID: 21501367.
16. Eckhart L, Lippens S, Tschachler E, Declercq W. Cell death by cornification. Biochim Biophys Acta. 2013;1833(12):3471-80. Epub 20130620. doi: 10.1016/j.bbamcr.2013.06.010. PubMed PMID: 23792051.
17. Muenzner P, Bachmann V, Zimmermann W, Hentschel J, Hauck CR. Human-restricted bacterial pathogens block shedding of epithelial cells by stimulating integrin activation. Science. 2010;329(5996):1197-201. doi: 10.1126/science.1190892. PubMed PMID: 20813953.
18. Edwards JL, Shao JQ, Ault KA, Apicella MA. Neisseria gonorrhoeae elicits membrane ruffling and cytoskeletal rearrangements upon infection of primary human endocervical and ectocervical cells. Infect Immun. 2000;68(9):5354-63. PubMed PMID: 10948165; PubMed Central PMCID: PMCPMC101799.
19. Merz AJ, Rifenbery DB, Arvidson CG, So M. Traversal of a polarized epithelium by pathogenic Neisseriae: facilitation by type IV pili and maintenance of epithelial barrier

function. Mol Med. 1996;2(6):745-54. PubMed PMID: 8972489; PubMed Central PMCID: PMCPMC2230138.

20. Merz AJ, So M. Interactions of pathogenic neisseriae with epithelial cell membranes. Annu Rev Cell Dev Biol. 2000;16:423-57. doi: 10.1146/annurev.cellbio.16.1.423. PubMed PMID: 11031243.

21. Edwards VL, Wang LC, Dawson V, Stein DC, Song W. Neisseria gonorrhoeae breaches the apical junction of polarized epithelial cells for transmigration by activating EGFR. Cell Microbiol. 2013;15(6):1042-57. Epub 2013/01/21. doi: 10.1111/cmi.12099. PubMed PMID: 23279089; PubMed Central PMCID: PMCPMC5584544.
